# Increased expression of CD133 and reduced dystroglycan expression are strong predictors of poor outcome in colon cancer patients

**DOI:** 10.1186/1756-9966-31-71

**Published:** 2012-09-11

**Authors:** Claudio Coco, Gian Franco Zannoni, Emanuele Caredda, Stefano Sioletic, Alma Boninsegna, Mario Migaldi, Gianluca Rizzo, Luca Reggiani Bonetti, Giannicola Genovese, Egidio Stigliano, Achille Cittadini, Alessandro Sgambato

**Affiliations:** 1 Dipartimento di Scienze Chirurgiche, Università Cattolica del Sacro Cuore, Rome, Italy; 2 Istituto di Anatomia Patologica, Università Cattolica del Sacro Cuore, Rome, Italy; 3 Istituto di Patologia Generale, Università Cattolica del Sacro Cuore, Largo Francesco Vito 1, Rome 00168, Italy; 4 Dipartimento Misto di Anatomia Patologica e di Medicina Legale, Sezione di Anatomia Patologica, University of Modena and Reggio Emilia, Modena, Italy

**Keywords:** Colon cancer, Dystroglycan, CD133, Cancer stem cell, Prognostic marker, Survival analysis

## Abstract

**Background:**

Expression levels of CD133, a cancer stem cell marker, and of the α-subunit of the dystroglycan (α-DG) complex, have been previously reported to be altered in colorectal cancers.

**Methods:**

Expression levels of CD133 and α-DG were assessed by immunohistochemistry in a series of colon cancers and their prognostic significance was evaluated.

**Results:**

Scattered cells positive for CD133 were rarely detected at the bases of the crypts in normal colonic mucosa while in cancer cells the median percentage of positive cells was 5% (range 0–80). A significant correlation was observed with pT parameter and tumor stage but not with tumor grade and N status. Recurrence and death from disease were significantly more frequent in CD133-high expressing tumors and Kaplan-Meier curves showed a significant separation between high vs low expressor groups for both disease-free (p = 0.002) and overall (p = 0.008) survival.

Expression of α-DG was reduced in a significant fraction of tumors but low α-DG staining did not correlate with any of the classical clinical-pathological parameters. Recurrence and death from the disease were significantly more frequent in α-DG-low expressing tumors and Kaplan-Meier curves showed a significant separation between high vs low expressor tumors for both disease-free (p = 0.02) and overall (p = 0.02) survival. Increased expression of CD133, but not loss of α-DG, confirmed to be an independent prognostic parameters at a multivariate analysis associated with an increased risk of recurrence (RR = 2.4; p = 0.002) and death (RR = 2.3; p = 0.003).

**Conclusions:**

Loss of α-DG and increased CD133 expression are frequent events in human colon cancer and evaluation of CD133 expression could help to identify high-risk colon cancer patients.

## Background

The cancer stem cell (CSC) model of tumorigenesis postulates that only a small number of cancer cells are able to both self renew and give rise to a differentiated progeny. CSC are believed to be responsible for the primary disease as well as its recurrence and metastasis. Thus, it is expected that their evaluation in clinical samples might provide useful information for a better prediction of disease aggressiveness and evolution. Although phenotypic characterisation of colon CSCs is still controversial, CD133 is presently considered a useful marker to identify CSC in colorectal cancers and its detection has been used to evaluate the prognostic significance of CSC in colon cancer patients [1-3].

Dystroglycan (DG) is a non-integrin adhesion molecule expressed in a wide variety of tissues at the interface between the basement membrane and the cell membrane [4]. It is formed by two subunits, the α (extracellular) and β (transmembrane) subunits which bind to the major ECM components and proteins involved in signal transduction and cytoskeleton organization, respectively. DG has been implicated in several cell functions (i.e., growth control, differentiation, shape change and movement) which are all relevant in the process of tumour development and metastasis [4-7]. We and others demonstrated that DG expression, and mainly α-DG, is reduced or lost in a variety of human cancer cell lines and primary tumours and overall, the available findings indicate that loss of DG expression is a frequent event in human malignancies and might play an important role in human tumour development and progression [4-6,8-10].

In the present study, CD133 and DG expression levels were analyzed by immunostaining in specimens of human primary colon cancers from a large group of patients with a long term follow-up and their relation with traditional prognostic indicators and with the clinical outcome of the patients was evaluated.

## Materials and methods

### Patient characteristics and tissue samples

Tissue specimens used for immunohistochemical analyses were obtained from a series of consecutive, unselected patients who had undergone curative surgery for colon cancer at the Division of Surgery, Policlinico “Agostino Gemelli”, School of Medicine, Università Cattolica del Sacro Cuore, Rome, Italy, from June 2000 to December 2003 and for whom clinicopathological data were available. A curative surgery was defined as one in which no macroscopic tumour remained at the end of surgery and in which histopathologic examination of the surgical specimen showed no tumour at the margins of resection. Distant metastases at the time of resection were excluded by preoperative liver ultrasonography and/or CT scan, chest X-ray and intraoperative exploration. After excluding cases with previous personal and/or familiar tumour history and patients with multiple colon cancers and multiple primary cancers or who received preoperative adjuvant therapy or were lost to follow-up, a cohort of 137 patients was selected for this study. Formalin fixed, paraffin embedded specimens were retrieved for this study from the archives of the Department of Pathology and two experienced pathologists (GFZ and MM) confirmed the histological diagnosis of each lesion. Histological tumour grading and staging were assessed according to standard criteria [11]. Proximal colon was defined as the large bowel proximal to the splenic flexure, and distal colon was defined as the large bowel distal to the splenic flexure excluding rectum. Treatment remained reasonably consistent during the study period.

### Immuno peroxidase detection of CD133 and α-DG

Immunohistochemical analyses were performed on routinely processed, formalin-fixed, paraffin-embedded tissues employing an avidin–biotin complex immunoperoxidase technique, as previously described [12,13]. A specific polyclonal anti-CD133 antibody (Santa Cruz Biotechnology, Santa Cruz, CA, USA; 1:100) was used for the staining. Comparable results but with a weaker staining were obtained using the monoclonal AC133 antibody (Miltenyi Biotec, Bergisch Gladbach, Germany; 1:10) (data not shown). The monoclonal anti α-DG antibody (clone VIA4-1) (Upstate Biotechnology, Lake Placid, NY) was used at a concentration of 10 μg/ml in PBS with 1% horse serum.

Controls for specificity of staining were performed by immunostaining duplicate sections in the absence of the primary antibody. Positive and negative control slides were included within each batch of slides.

All scoring and interpretations of the results were made by two of the authors independently (MM and LR) without knowledge of other clinicopathological variables. To assess interobserver variation, the results of the two measurements were compared by paired *t* test and no statistical differences were found (data not shown). The few cases with discrepant scoring were re-evaluated jointly on a second occasion, and agreement was reached.

### Statistical analysis

The association between molecular and clinic-pathological parameters were calculated using contingency table methods and tested for significance using the Pearson’s chi-square test. Patients were all uniformly followed-up at our Institution and disease free survival (DFS) was defined as the interval between surgery and the first documented evidence of disease in local-regional area and/or distant sites. Overall survival was defined as the interval between surgery and death from the disease. Patients who died for causes unrelated to disease were not included in the survival analyses. All calculations were performed using the STATA statistical software package (Stata Corporation, College Station, Texas) and the results were considered statistically significant when the p value was ≤0.05.

## Results

### Clinicopathological findings

The clinicopathological findings of the 137 patients are listed in Table 1. The median age of the patients was 68 years (range, 31–86 years; mean, 66.8), and they included 78 males (mean age 68.20 ± 10.10 ) and 59 females (mean age 64.96 ± 12.60). According to TNM stage, 25 cases were stage I, 43 stage II and 69 stage III. Stage IV patients were excluded from the analysis. The pathological diagnosis was adenocarcinoma not otherwise specified (NAS) in 122 cases and mucinous adenocarcinoma in the remaining 15 cases. Based on grading, adenocarcinomas were classified as well- or moderately differentiated in 95 cases, and poorly differentiated in 42 cases.

**Table 1:** Clinicopathological data

**Age: 66.8 ±11.3 (mean age ± SD, year)**	
**Characteristics**	**No. of patients (%)**
**Gender**	
Male	78 (56.9)
Female	59 (43.1)
**Histotype**	
ADK NAS§	122 (89.1)
Mucinous	15 (10.9)
**Tumour location**	
Proximal	60 (43.8)
Distal	77 (56.2)
**Grading**	
Well	9 (6.6)
Modertae	86 (62.8)
Poor	42 (30.7)
**TNM**	
T1	12 (8.8)
T2	17 (12.49
T3	101 (54.7)
T4	7 (24.1)
**Nodal status**	
N0	76 (55.5)
N+	61 (45.5)
**Tumor stage**	
I	25 (18.2)
II	43 (31.4)
III	69 (50.4)
**Recurrence**	
Yes	57 (41.6)
Not	80 (58.4)
**Follow-up**	
Deceased	51 (37.2)
Alive	86 (62.8)

**§** ADK NAS: adenocarcinoma not otherwise specified.

### CD133 expression is increased in colon carcinomas and correlates with the clinical outcome of patients

**Figure 1:**
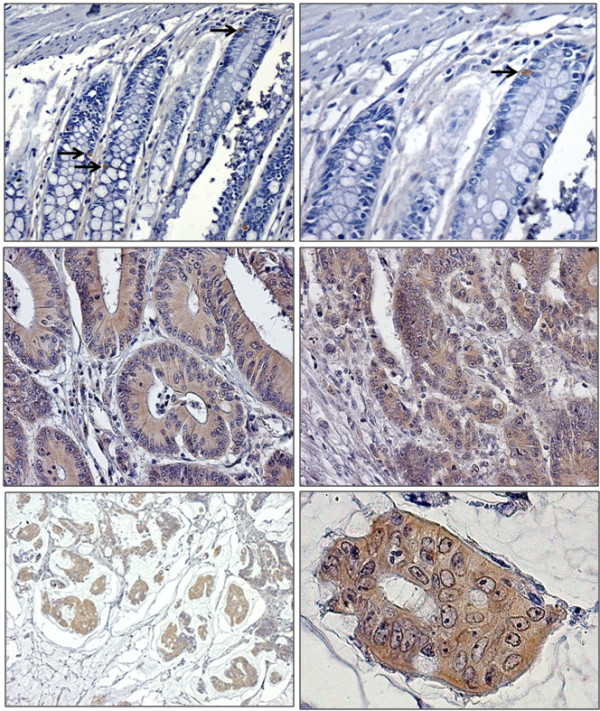
**Examples of CD133 immunohistochemical staining in human colon samples. **(**A** and **B**) Normal colonic mucosa. Note the rare (→) positivity for CD133 (A, × 200 and B, × 400). (**C**) A early dysplastic lesion of colon tumorigenesis showing a marked positive immunostaining for CD133 (× 200). (**D**) Example of a moderately differentiated NAS adenocarcinoma displaying a diffuse staining for CD133 (× 200). (**E **and **F**) Examples of mucinous poorly differentiated adenocarcinomas displaying a strong and diffuse cytoplasmic staining for CD133 with a clear immuno-negativity of nuclei (× 200 and × 550).

CD133 expression was evaluated by immunostaining in a series of 137 primary human colon cancers (Table 1) and only a clear staining of the cell membrane and/or cytoplasm was regarded as positive. Normal colonic mucosa was present in about 50% of the cases and scattered positive cells were rarely detected at the bases of the crypts (Figure 1A and B).

In cancer cells the median percentage of positive cells was 5% (range 0–80; mean = 13%) and CD133 staining was not detectable in tumour cells in 30 out of 137 (22%) specimens (Figure 1C-F). When cases were stratified according with pT parameter, median percentage of positive cells was 17.5 (range 0–70; mean = 24%), 10.0 (range 0–60; mean = 16), 2.0 (range 0–65; mean = 9) and 10 (range 0–80; mean = 13) in pT1, 2, 3 and 4 tumours, respectively, and these differences were significant (p = 0.02). Moreover, using the 5% positive cells as cut-off to distinguish between high (>5%) and low (≤5%) staining, high CD133 staining was detected in 9 (75%) of the 12 pT 1 cancers and in 10 (59%), 27 (36%) and 19 (58%) of the pt2, pT3 nd pT4 cancers, respectively and cross-tab analysis identified a significant correlation (p = 0.02) between the two parameters (Table 2). Significance was also evident when earlier (pT1-2) tumours (66%) were compared together vs more advanced (pT3-4) (42.6%) cancers (p = 0.02). No correlation was observed with either tumour grade and N status.

**Table 2:** CD133 expression in relation to clinical and pathological parameters in a series of 137 colon cancers

	**Total**	**Low**	**High**	***p*value**
		**n (%)**	**n (%)**	
**Gender**				
Males	78	41 (53)	37 (47)	
Females	59	31 (52)	28 (48)	n.s.
**Age (yr)**				
≤68	73	35 (48)	38 (52)	
>68	64	37 (58)	27 (42)	n.s.
**Tumor Grading**				
1	9	4 (44)	5 (56)	
2	86	50 (58)	36 (42)	
3	42	18 (43)	24 (57)	n.s.
**pT parameter**				
pT1	12	3 (25)	9 (75)	
pT2	17	7 (41)	10 (59)	
pT3	75	48 (64)	27 (36)	
pT4	33	14 ( 42)	19 (58)	0.02
**Nodal status**				
Negative	76	42 (55)	34 (45)	
Positive	61	30 (49)	31 (51)	n.s.
**Tumor stage**				
I	25	9 (36)	16 (64)	
II	43	31 (72)	12 (28)	
III	69	32 (46)	37 (54)	0.006
**Recurrence**				
YES	57	22 (39)	35 (61)	
NOT	80	50 (62)	30 (37)	0.005
**Follow-up**				
Deceased	51	20 (39)	31 (61)	
Alive	86	52 (61)	34 (39)	0.013
**α-DG staining**				
Low	68	28 (41)	40 (59)	
High	69	44 (64)	25 (36)	0.006

n.s.: not significant.

On the other hand, high CD133 staining was detected in 16 (64%) of the 25 stage 1, 12 (28%) of the 43 stage II and in 37 (54%) of the 69 more advanced stage 3 cancers and cross-tab analysis identified a significant correlation (p = 0.006) between the two parameters (Table 2). Significance was no longer evident when stage 1/2 cancers (41%) were compared overall with more advanced stage 3 cancers (54%) (p = 0.09) (Table 2).

When CD133 staining was analyzed in relation with clinical outcome, both disease recurrence and disease-related death were more frequent in patients whose tumor expressed a high staining for CD133. Overall, median percentage of positive cells was 1.0 (range 0–80; mean = 12.3 ± 19.5%) and 10.0 (range 0–80; mean = 13.9 ± 14.8%) in non recurrent and recurrent cases, respectively, but this difference was not significant. When tumours were stratified according with CD133 expression, median DFS of CD133 low expressor tumors was longer compared to high expressor cases (80.5 ± 36.8 vs 48.0 ± 39.1 months) and this difference was significant (p = 0.001). Moreover, when tumours were stratified according with CD133 expression, twenty-two (30.6%) out of 72 low expressor cases and 35 (54%) among the remaining 65 cases recurred during the period of follow-up and this difference was significant (p = 0.005) as also confirmed by the Kaplan-Meier curves of DFS which displayed a significant separation between the two groups of patients (p = 0.002 by log-rank test) (Figure 3A). Similarly, thirty-one (47.7%) out of 65 patients with high expresssor tumours and only 20 (27.8%) of the 72 remaining ones died of disease during the period of follow-up and this difference was significant (p = 0.013) although median percentage of positive cells was 2.0 (range 0–80; mean = 13.6 ± 21.0%) and 10.0 (range 0–40; mean = 12.0 ± 10.0%) in alive and death patients, respectively, and this difference was not significant. Thus, patients with tumors displaying a higher staining for CD133 were more likely to die for the disease compared with low expressor tumors as confirmed by the Kaplan-Meier curves which displayed a significant separation between the two groups of patients (p = 0.008 by log-rank test) (Figure 3B). Hence, increased expression of CD133 was associated with an increased risk of recurrence and death in our series of colon cancers (Figures 3A and B).

**Figure 2:**
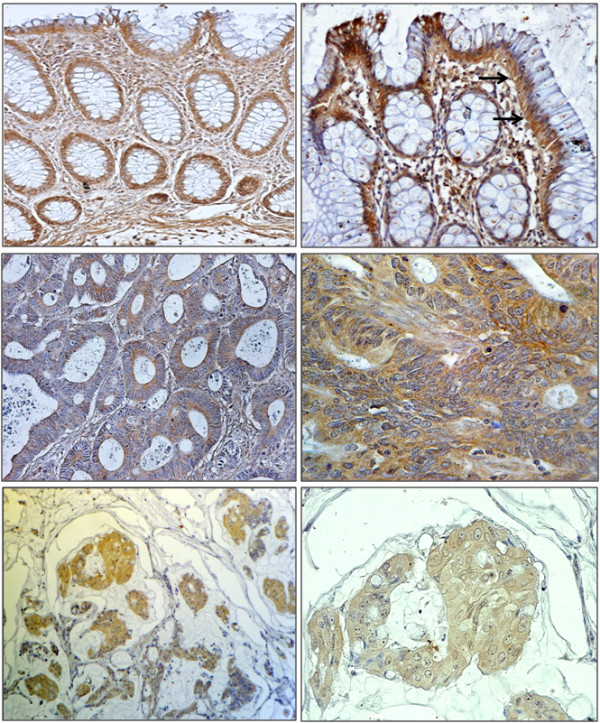
**Examples of α-DG immunohistochemical staining in human colon samples. **(**A**) Normal colonic mucosa. Note the intense cytoplasmic immunopositivity of caliciform cells of the cryptes (× 20) and the positive staining of the stroma likely due its muscolar fraction, which served as positive control. (**B**) Normal colonic mucosa. Note the strongest staining on the basis of cells and the reinforcement of basal membrane (arrows) (× 40. (**C**) A well differentiated NAS adenocarcinoma displaying a diffuse staining for α-DG (× 200). (**D**) A poorly differentiated NAS adenocarcinoma displaying an intense cytoplasmic staining for α-DG (× 400). (**E **and **F**) A mucinous poorly differentiated adenocarcinoma displaying a clear diffuse cytoplasmic staining for α-DG (× 200 and × 550).

**Figure 3:**
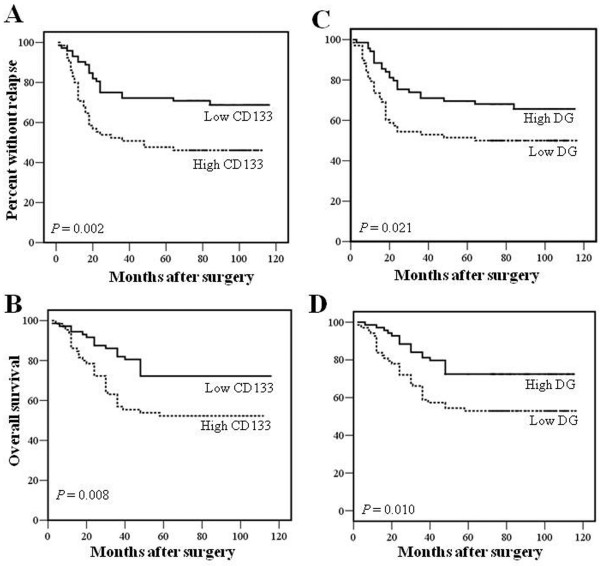
**Kaplan-Meier curves for disease-free (*upper panels*) and overall (*lower panels*) survival in a series of 137 colorectal cancer patients. **Patients were stratified by CD133 expression (**A, B**) or according to the level of α-DG expression (**C, D**) (see text for details).

### Loss of α-DG expression is a frequent event in colon cancers and correlates with the clinical outcome of patients

We previously reported that α-DG expression, as assessed by western blot analysis, is frequently lost in human colon cancer cell lines and primary tumours and correlates with tumour grade and stage. In this study, α-DG expression level was assessed by immunostaining in the same series of colon cancer samples using a specific anti- α-DG antibody (Figure 2). An evident staining was observed in the majority of normal specimens (Figure 2A and B). In tumour tissues staining was highly heterogeneous in term of percent of positive cells with the median percentage of positive cells being 30% (range 0–90; mean = 35%) (Figure 2C-F). DG levels did not correlate with most of the analyzed parameters (age, gender, pT parameter, tumour stage, grading, N status) (Table 3). As previously mentioned, low DG expression was also more frequent in tumours expressing increased levels of CD133 (p = 0.006) (Table 2).

**Table 3:** α-DG expression in relation to clinical and pathological parameters in a series of 137 colon cancers

	**Total**	**Low**	**High**	***p*value**
		**n (%)**	**n (%)**	
**Gender**				
Males	78	42 (54)	36 (46)	
Females	59	26 (44)	33 (56)	n.s.
**Age (yr)**				
≤68	73	33 (45)	40 (55)	
>68	64	34 (54)	29 (46)	n.s.
**Tumor Grading**				
1	9	3 (33)	6 (67)	
2	86	45 (52)	41 (48)	
3	42	20 (48)	22 (52)	n.s.
**pT parameter**				
pT1	12	7 (58)	5 (42)	
pT2	17	7 (41)	10 (59)	
pT3	75	35 (47)	40 (53)	
pT4	33	19 (58)	14 (42)	n.s.
**Nodal status**				
Negative	76	37 (49)	39 (51)	
Positive	61	31 (51)	30 (49)	n.s.
**Tumor stage**				
I	25	11 (44)	14 (56)	
II	43	18 (42)	25 (58)	
III	69	39 (56)	30 (44)	n.s.
**Recurrence**				
YES	57	34 (60)	23 (40)	
NOT	80	34 (42)	46 (58)	0.035
**Follow-up**				
Deceased	51	32 (63)	19 (37)	
Alive	86	36 (42)	50 (58)	0.014

n.s.: not significant.

When DG staining was analyzed in relation with clinical outcome, low DG expression was more frequent in recurrent vs non-recurrent cases (p = 0.035) but the median percentage of positive cells was not different between the two subgroups of patients. Finally, low DG expression was also more frequent in deceased vs alive patients (p = 0.014) and the median percentage of positive cells tended to be lower in deceased (median = 30.0; range 0–80; mean = 31.1%) compared to surviving patients (median = 40.0; range 0–90; mean = 38.4%) (p = 0.07). When tumours were stratified according with DG expression, mean DFS of DG low expressor tumors was shorter compared to high expressor cases (65.8 vs 84.4 months) and this difference was significant (p = 0.035) as also confirmed by the Kaplan-Meier curves of DFS which displayed a significant separation between the two groups of patients (p = 0.02 by log-rank test) (Figure 3C). Similarly, mean OS of DG low expressor tumors was shorter compared to high expressor cases (72.6 vs 91.8 months) and this difference was significant (p = 0.025) as also confirmed by the Kaplan-Meier curves of OS which displayed a significant separation between the two groups of patients (p = 0.01 by log-rank test) (Figure 3D).

### CD133 immunostaining is an independent prognostic parameter in colon cancer patients

To further explore the prognostic significance of CD133 and α-DG, we built a Cox regression model including the two parameters with tumour grade, pT and N parameters. Only a high CD133 staining (*p* = 0.002; C.I. 1.365-4.171; RR = 2.4) and lymph node involvement (*p* = 0.001; CI = 1.532-5.876; RR = 3.0) confirmed to be independent predictors of shorter disease-free survival (Table 4). It is noteworthy that α-DG confirmed to be an independent prognostic indicator when CD133 was not included in the model (*p* = 0.024; C.I. 1.086-3.144; RR = 1.8), a result expected given the correlation between the two parameters.

**Table 4:** Contribution of various potential prognostic factors to disease free survival by Cox regression analysis in colon cancer patients

	**Hazard**	**95% confidence**	
**Variable**	**ratio**	**interval**	***p*value**
Tumor grade*	1.438	0.801-2.583	0.223
pT parameter#	2.027	0.806-5.094	0.133
Node status**	3.000	1.532-5.876	0.001
CD133§	2.386	1.365-4.171	0.002
Dystroglycan§§	1.629	0.950-2.794	0.076

The risk ratio is given as: * higher (G3) versus lower grade (G1/2); # higher (pT3/4) versus lower (pT1/2) pT parameter; ** node-positive vs node-negative; § positive vs negative and §§ negative vs positive.

A similar Cox regression model including also the age confirmed the independent prognostic significance of only CD133 staining (*p* = 0.003; C.I. 1.332-4.114; RR = 2.3) and lymph node involvement (*p* = 0.001; CI = 1.546-5.911; RR = 3.0) also in term of overall survival (Table 5). α-DG staining did not display an independent prognostic significance also when CD133 was not included in the model (*p* = 0.051; C.I. 0.997-2.902; RR = 1.7).

**Table 5:** Contribution of various potential prognostic factors to overall survival by Cox regression analysis in colon cancer patients

	**Hazard**	**95% confidence**	
**Variable**	**ratio**	**interval**	***p*value**
Age°	1.431	0.842-2.432	0.185
Tumor grade*	1.380	0.767-2.484	0.282
pT parameter#	1.850	0.744-4.599	0.185
Node status**	3.023	1.546-5.911	0.001
CD133§	2.341	1.332-4.114	0.003
Dystroglycan§§	1.462	0.845-2.532	0.175

The risk ratio is given as: ° older (>68 y) versus younger patients; * higher (G3) versus lower grade (G1/2); # higher (pT3/4) versus lower (pT1/2) pT parameter; ** node-positive vs node-negative; § positive vs negative and §§ negative vs positive.

## Discussion

In this study, the expression of the surface markers CD133 and α-DG was evaluated in a subset of colon cancers and their potential prognostic significance was investigated.

We and others previously reported that loss of the α subunit of the DG complex (α-DG) is a frequent event in human cancers [6,8,10,12,14-16]. We also demonstrated, by western blot analysis, that α-DG is frequently reduced in colon cancer tissues compared to normal adjacent normal tissues while the β subunit did not display significant variations between normal and tumour tissues [12]. In this study, we further analyzed the DG involvement in colon tumorigenesis and confirmed, by immunohistochemistry, that detection of α-DG is frequently reduced or lost in the majority of cancer tissues compared to adjacent normal tissues (Figure 2) and that loss of α-DG correlates with a worse prognosis (Figure 3). These findings are in agreement with the proposed tumour-suppressor function of the protein [17] and with previous observations in several human malignancies [5,18-20]. The functional inactivation of the DG complex in tumour cells has been mainly attributed to post-translation mechanisms which cause the loss and/or an altered glycosylation of the extracellular α-DG [21-25]. Since DG subunits are encoded by a single gene and are formed upon cleavage of a precursor protein [6,26], our previous findings that β-DG subunit is detectable in most of the colon cancers in which α-DG was not detectable [12] suggest that, as reported in other types of human malignancies, this lack of detection is likely not due to loss of gene expression but to a specific posttranscriptional mechanism affecting α-DG processing in colon cancer cells.

The DG complex connects the ECM network to the cytoskeleton and is likely involved in the regulation of signaling pathways [6]. Thus, regardless of the underlying molecular mechanisms, loss of a functional α-DG subunit can play an important role in the tumorigenesis process by compromising the formation of strong contacts between ECM and the cytoskeleton of cells resulting, as for integrins, in less sticky tumour cells able to move unhindered in the extracellular matrix, thus predisposed to invade surrounding tissue and metastasize [6,17]. It will be of interest to evaluate DG expression in the entire process of human colon tumorigenesis (i.e., from early to metastatic lesions).

CD133 has been reported to be a CSC marker in colorectal cancer [27,28], and, although some doubts have been arisen about its ability to specifically identify tumour-initiating cells [29], it has been widely used to identify and analyze CSC in colorectal cancers. We were able to detect CD133 staining in the majority (78%) of colon cancers analyzed although with a high heterogeneity in term of percentage of positive cells (range 0-80%) whose increase was associated with an increased risk of recurrence and death for the disease (Table 2 and Figure 3). These findings are in agreement with previous evidence suggesting a potential prognostic role of the protein in colon cancer patients. Indeed, it has been reported that CD133 expression levels correlate with patients survival in colorectal cancers [1-3,30,31] although available data on the presence of CD133+ cells in human colorectal cancers are not always consistent in term of distribution and percentage of positive cells. Several factors might explain such discrepancies: i) inadequate patient cohort; ii) mixed tumour stages; iii) different criteria used to identify positive staining; iv) different cut-off used to discriminate positive and negative tumours; v) different antibodies used for the analysis, with the latter being, in our opinion, the most important factor. Different antibodies have been indifferently used in different studies for the detection of the CD133 molecule. In our opinion this can be a highly confusing factor. Indeed, we previously demonstrated, by western blot analysis, that CD133 is expressed at various levels in colon cancers [32,33] and that different results can be obtained by using different antibodies [34] and similar observations have been also reported by other Authors [35,36]. The observation that high CD133 expression has been reported to be a negative prognostic factor for colorectal cancers in several studies using different antibodies strongly suggests an important prognostic significance of its detection [1,2,37]. In our study, CD133 also confirmed to be an independent risk factor for a shorter disease-free and overall survival in a multivariate analysis (Tables 4 and 5). These findings are consistent with similar results reported in other human cancers and warrant studies on larger cohorts of patients to further evaluate its suitability as a prognostic marker in the clinical management of colon cancer patients.

We observed an unexpected behaviour of CD133 expression which tended to be higher in the lowest grade/stage tumours than in more advanced lesions. Although not expected, this distribution is consistent with previous findings in a mouse model of colon carcinogenesis [38] and in human primary colon cancers [39]. Indeed, in mouse colon carcinogenesis we observed a significantly increased expression of CD133, assessed by immunohistochemistry, in early neoplastic lesions which tended to decrease with tumour development, although remaining always higher in cancer than in normal adjacent tissues [38] and an increased CD133 expression, assessed using a quantitative reverse-transcription PCR, was reported in Dukes A compared to Dukes B and C colon cancers [39]. These findings are in agreement with the proposed ability of the protein to specifically identify tumour initiating cells, important for the growth of both primary and recurrent/metastatic cancers [40] and thus mainly involved in the most active phases of tumour development, i.e., in early lesions (low grade and low stage cancers) as well as in metastatic lesions. Consistent with this hypothesis, CD133 expression has been reported to be highly expressed in colon cancers with early liver metastases and to be a potential biomarker for the early liver metastases [41] and we also previously reported an increased percentage of CD133+ cells, assessed by flow cytometry, in metastatic vs primary colon cancers, [42]. It will be of interest to evaluate the immunohistochemical CD133 expression in the entire process of human colon tumorigenesis (i.e., from early to metastatic lesions) and evaluate how it correlates with tumour development.

An unexpected finding of the present study was the observed inverse relationship between CD133 and α-DG expression (Table 2). The significance of this observation is unknown since no data are available up to date linking the two molecules. It is of interest that DG expression increases with cell differentiation while CD133 expression decreases in differentiated cells [7,33,43-45] thus suggesting a potential functional link between the two molecules. Further studies will be required to fully understand the biological significance of the observed relationship between the two molecules.

## Conclusions

To our knowledge, this is the first study analyzing the immunohistochemical expression of both CD133 and α-DG, two surface molecules previously reported to be altered in human colorectal cancers, in a large series of colon cancer patients. Our results demonstrate that an inverse relationship exists between the two molecules (Table 2) and that CD133 expression is an independent risk factor associated with patient survival in multivariate analyses (Tables 4 and 5). The role of CD133 as a biomarker for CSC is still debated [46]. Regardless of its significance as a CSC marker, however, our results suggest that evaluation of CD133 staining might be useful to identify colon cancer patients at high risk of recurrence and death. Thus, we believe, as previously reported, that it will be important to define standardized procedures and reagents to evaluate expression of this molecule in clinical samples [34]. Afterwards, a prospective multicenter evaluation of CD133 immunostaining on a larger population of surgically resected colon cancers is warranted to allow a conclusive and definitive assessment of its suitability in predicting tumor aggressiveness and outcome in colon cancer patients.

## Competing interests

The authors declare that they have no competing interests.

## Authors' contributions

CC, AC, AS conceived the study and participated in its coordination. CC, GFZ, MM, AS participated in protocol design. GFZ, SS, MM, LRB provided tissue samples. ET prepared the tissue slides. AB, EC performed the immunohistochemical assays. SS, MM, LRB evaluated and scored the staining. CC, GR, GG provided clinical information. MM, AS performed statistical analyses and drafted the manuscript. All authors read and approved the manuscript.
